# CASE REPORT Temporomandibular Joint Arthroplasty With Human Amniotic Membrane: A Case Report

**Published:** 2013-03-18

**Authors:** Florian Bauer, Lukas M. Hingsammer, Klaus-Dietrich Wolff, Marco R. Kesting

**Affiliations:** Department of Oral and Maxillofacial Surgery, Technical University of Munich, Klinikum Rechts der Isar, Ismaninger Str 22, D-81675 Munich, Germany

## Abstract

This case reports the usage of human amniotic membrane combined with a costochondral graft as an interpositional material in temporomandibular joint reconstruction for the first time in humans. Because of the favorable outcome 20 months postoperatively, it has to be considered as an approach bringing to light the antiadhesive potential of amniotic membrane. This case report must be regarded as initial spadework and should motivate other institutions to intensify their clinical research in this field. Because of the fact that currently used interpositional materials do not prevent the recurrence of temporomandibular joint ankylosis sufficiently, it is of great interest to establish a proper therapeutic intervention fulfilling these demands. Furthermore, the demonstrated antiadhesive properties of amniotic membrane highlight its multifaceted field of application. Nevertheless, further studies have to prove the findings reported in our case.

Gap arthroplasty and interpositional arthroplasty are the most commonly used therapeutic options to treat TMJ (temporomandibular joint) ankylosis. The major complication of both interventions is the recurrence of ankylosis. To date, no surgical or nonsurgical procedure can guarantee nonrecurrence; therefore, its effective therapy posts a clinical challenge. On the basis of the unsatisfactory results experienced with the current treatment options, our aim is to find an adequate new way to deal with TMJ ankylosis.^1^^-^5

## THE CASE

A 53-year-old, otherwise healthy woman presented herself because of pain in the left TMJ region and an insufficient mouth opening, which exhibited itself in the preceding weeks. Clinical examination revealed a maximum interincisal opening of 15 mm. In preliminary investigations, radiographic, computed tomographic, and magnet tomographic analyses were performed and a neoplasm at the left condylar process, involving the whole joint cavity, was detected. To clarify the finding, a biopsy of the suspicious mass was performed and a giant cell tumor was diagnosed. After careful evaluation of the diagnosis, risks, and benefits of all possible treatment options, the woman was assessed to undergo radical excision of the tumor, including total resection of the condylar process plus the articular disk. On the basis of the positive outcome of the previously performed animal experiments and the informed consent of the patient, a costochondral graft combined with an allogeneic human amniotic membrane (HAM), was chosen to reconstruct the joint head. For this intention, ethical approval of the independent ethics committee was obtained.

## METHODS

**Figure 1:**
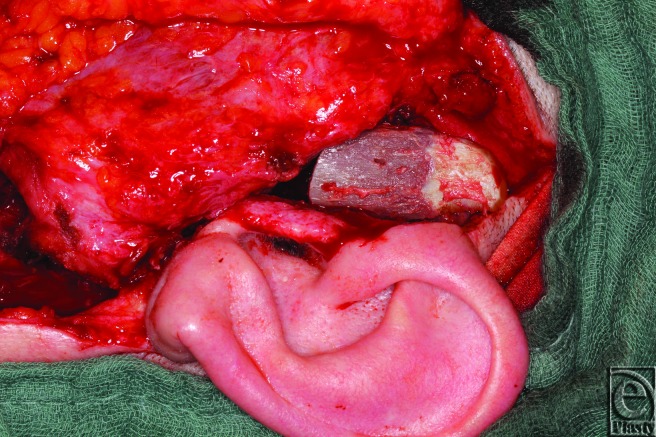
Positioning of the harvested rib graft.

**Figure 2:**
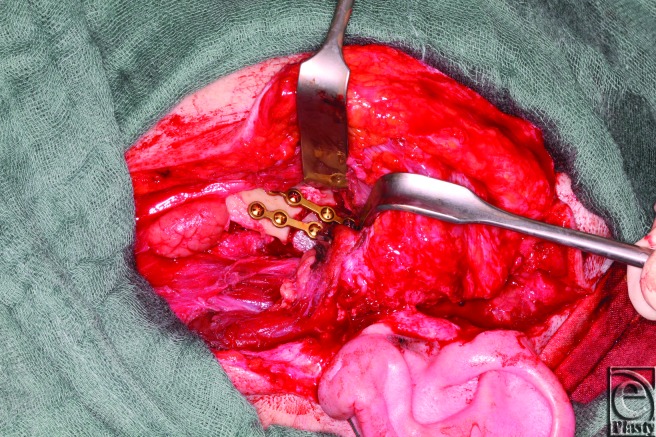
Osteosynthesis of the rib graft with 2 mini plates.

**Figure 3:**
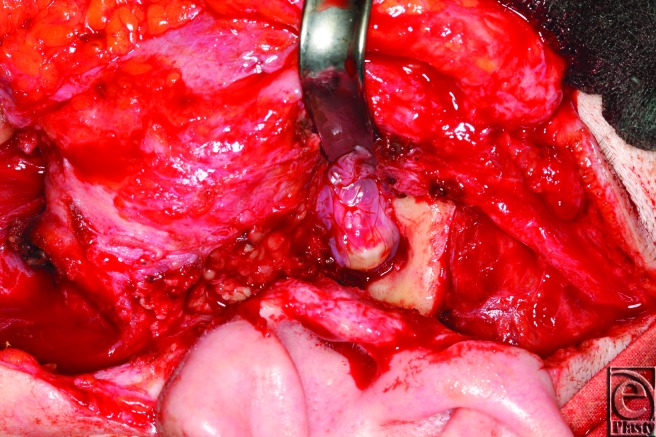
Insertion of the amniotic membrane.

Starting the operation, the occlusion was fixed with wire splints. In the next step, the costochondral graft was harvested without any complication from the right ninth rib and kept in a moist bandage during excision of the neoplasm. Through a submandibular and a preauricular approach, the tumor and the condylar process were totally resected. The margins of all frozen sections were clear. The harvested autologous costochondral rib graft was prepared and shaped before transplantation (Fig 1). Osteosynthesis of the cleaned, shaped, and adjusted rib graft to the mandibular ramus was ensured with 2 mini plates (Fig 2). The chondral part of the rib graft was placed facing the articular fossa of the temporal bone, forming a new condyle that ensured articular functions. The cryoconserved and moist allogeneic amniotic membrane was mounted onto the condylar part of the new joint head. Thereby, the epithelial side of the membrane was adjusted toward the joint cavity (Fig 3). Before closing the operation site, the patient's occlusion was reevaluated and adjusted. Intraoperatively, no surgical complications were noticed and also the anesthesia was well tolerated by the patient.

## RESULTS

**Figure 4:**
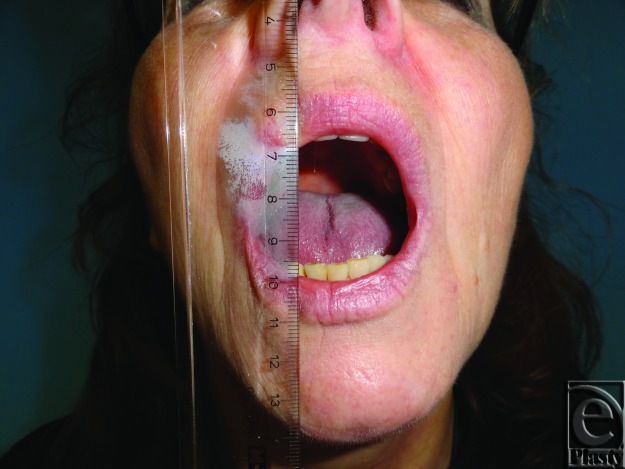
Maximum interincisal mouth opening, 8 months postoperatively.

The postoperative treatment included intensive physiotherapy and regular follow-up appointments. Wound healing, mouth opening, and her general condition were evaluated on the 1st, 3rd, 5th, 7th, 14th, and 30th day and then every month postoperatively. A 20-month follow-up of the patient showed uneventful wound healing without complication. Ankylosis of the TMJ was avoided by long-term observation and the patient presented a maximum interincisal opening of 32 mm 8 months postoperatively (Fig 4). The correction of the ankylosis has to be considered as the consequence of an optimal interplay of the resection of the tumor, the successful reconstruction, aggressive physiotherapy, and good compliance by the patient.

## DISCUSSION

Temporomandibular joint ankylosis is classified into true and false ankylosis. False ankylosis is the restricted movement of the joint due to extra-articular factors such as muscles, nerves, or the coronoid process. True or intra-articular TMJ ankylosis is the obliteration of the TMJ by fibrous tissue, mainly resulting from trauma, surgical intervention, infection, rheumatoid arthritis, or neoplasm, causing pain, insufficient mouth opening, and difficulty in mastication and speech.^1^ The main treatment options for TMJ ankylosis are interposition arthroplasty and gap arthroplasty. There is some controversy in the literature regarding the most appropriate therapeutic intervention to use. Many systematic reviews tried to summarize the literature and to point out the best operative intervention, but no common consent could be established.^2^^,^^3^^,^^5^ In gap arthroplasty, the condylar process is cut and no interpositional material is inserted.^2^^,^^3^ The complications of gap arthroplasty are anterior open-bite deformities, functional impairment, and frequently reankylosis. In interpositional arthroplasty, alloplastic or biological materials are transplanted between the cut mandibular head and the joint cavity.^1^^,^^3^ Alloplastic materials, such as silicone or Teflon, achieve a preservation of the vertical height of the ramus, though allergic and foreign body reactions have been reported.^6^ The facts that alloplastic materials are very expensive and must not be used in children, limit their utilization.^7^ The disadvantage of biological materials such as fascia lata and fat graft is that they cannot preserve the vertical height of the mandibular ramus and in consequence, functional problems are common.^7^ Beside this, Alloderm, an acellular dermal matrix, showed good results when used for TMJ reconstruction.^8^ At present, the most frequently used interpositional materials are bone grafts such as metatarsal, sternoclavicular, or costochondral grafts.^9^ Among these, the costochondral graft interposition has been extremely popularized.^9^ In consequence, the use of an autologous costochondral graft combined with free fat graft, as it is reported to have the best long-term outcome, is considered the criterion standard.^6^^,^^10^^,^^11^ Nevertheless costochondral grafts have an unpredictable growth potential and their extraction is always accompanied by donor site morbidity.^12^^,^^13^ Furthermore, development and recurrence of TMJ ankylosis poses a problem with any interpositional material.^14^ In conclusion, none of the currently utilized methods can prevent TMJ ankylosis and leads to completely satisfactory joint reconstruction. Out of these insufficient therapeutic possibilities, it is our aim to establish a new therapy option to handle TMJ ankylosis. Amniotic cells have been documented to express various surface markers associated with embryonic stem cells and are known to have pluripotent properties.^15^^,^^16^ Production of anti-inflammatory factors, such as IL-1 and IL-2 receptor antagonists and IL-10, plus endostatin, all of which inhibit endothelial cell proliferation, angiogenesis, and tumor growth, is another property of amniotic cells.^17^ Because of these properties, amniotic cells were used in the form of amniotic membranes for skin transplantation for the first time in 1910 and nowadays many disciplines are trying to use the beneficial effects of amniotic cells.^18^^,^^19^ As HAM promotes wound healing and proven antiadhesive effects, it is used as a skin application in many surgical disciplines and in gynecology to prevent intrauterine adhesions.^10^^,^^20^^,^^21^ Concerning oncology, HAM is stated to interfere with tumor angiogenesis, growth, and metastasis. Its best known, most auspicious, and the only routinely accepted application in Germany is ocular surface reconstruction.^10^ As HAM has been found to prevent fibrosis and to promote wound healing, it is considered to have the potential to decrease or even prevent TMJ ankylosis.^19^^,^^22^^,^^23^ In the rabbit model, HAM has prevented fibrosis and reankylosis as an interpositional material.^1^ The aim of this case was to build on the encouraging outcome of the animal study and to prove these findings. Because of the fact that we used HAM combined with a costochondral graft, the results cannot be directly linked to HAM. On the basis of our experience, the outcome was better than in our conventionally treated cases, but no solid evidence exists. Although no fibrotic intergrowth, an uneventful integration of HAM, and recovery of a normal mouth opening range can be reported in this case, this innovative therapy option has to be compared to the currently applied interventions. As this case describes the usage of HAM combined with a costochondral graft in TMJ reconstruction for the first time, it has to be considered as an approach bringing to light the great potential of amniotic membrane in this indication. Beyond a doubt, one case makes it hard to accept efficacy and safety. Nevertheless, the reported antiadhesive effect, preventing TMJ ankylosis 20 months postoperatively, gives hope to finding a proper interpositional material preventing TMJ ankylosis. In any event, further clinical studies with high validity need to be performed to verify these findings.
